# Approximate Solution of Urysohn Integral Equations Using the Adomian Decomposition Method

**DOI:** 10.1155/2014/150483

**Published:** 2014-01-20

**Authors:** Randhir Singh, Gnaneshwar Nelakanti, Jitendra Kumar

**Affiliations:** Department of Mathematics, Indian Institute of Technology Kharagpur, Kharagpur 721302, India

## Abstract

We apply Adomian decomposition method (ADM) for obtaining approximate series solution of Urysohn integral equations. The ADM provides a direct recursive scheme for solving such problems approximately. The approximations of the solution are obtained in the form of series with easily calculable components. Furthermore, we also discuss the convergence and error analysis of the ADM. Moreover, three numerical examples are included to demonstrate the accuracy and applicability of the method.

## 1. Introduction

Nonlinear integral equations arise very frequently many branches of science and engineering such as biology, applied mathematics, physics, and many areas of analysis. We consider the following nonlinear Urysohn integral equation:

(1) $$\begin{array}{c} y\left(x\right)=f\left(x\right)+\overset{}{\underset{\Omega }{\int }}K\left(x,t,y\left(t\right)\right)dt,\;a\leq x\leq b. \end{array}$$

Depending on *Ω* = (*a*, *x*) or *Ω* = (*a*, *b*), (1) is named a nonlinear Volterra integral equation or a nonlinear Fredholm integral equation, respectively. We assume that the problem (1) has a unique solution. The following conditions are assumed:

(*C*_1_) *f*(*x*) is continuous;
(*C*_2_) the kernel *K* (*x*, *t*, *y*(*t*)) is continuous on *D* = {*Ω* × *Ω* × ℝ};
(*C*_3_) let the kernel *K* (*x*, *t*, *y*(*t*)) satisfy the Lipschitz condition
  (2) |K(x,t,y)−K(x,t,y∗)|≤L|y−y∗|,∀(x,t,y),(x,t,y∗)∈D,  

where *L* is Lipchitz constant.

Recently, a great deal of interest has been focused in the study of nonlinear Urysohn integral equation [1–8] and many of the references therein.

The objective of this paper is to apply the ADM for obtaining approximate series solution of the nonlinear Urysohn integral equations. Moreover, the convergence and error analysis of the ADM are discussed. Finally, we compare our numerical results with those obtained by [5].

## 2. Adomian Decomposition Method 

In the recent past, a lot of researchers [9–16] have expressed their interest in the study of ADM for various scientific models. Adomian [12] asserted that the ADM provides an efficient and computationally worthy method for generating approximate series solution for a large class of differential as well as integral equations. In order to apply ADM to Urysohn integral equation, we rewrite (1) as

(3) $$\begin{array}{c} y\left(x\right)=f\left(x\right)+\overset{b}{\underset{a}{\int }}N\left(y\right)dt,\;a\leq x\leq b, \end{array}$$

where *N* (*y*) = *K* (*x*, *t*, *y*(*t*)) is a nonlinear operator.

We now decompose the solution *y* (*x*) by series as

(4) $$\begin{array}{c} y\left(x\right)=\overset{\infty }{\underset{j=0}{\sum }}y_{j}\left(x\right), \end{array}$$

and the nonlinear functions *N* (*y*) is decomposed by series

(5) $$\begin{array}{c} N\left(y\right)=\overset{\infty }{\underset{j=0}{\sum }}A_{j}, \end{array}$$

where *A*_*j*_, *j* ∈ *ℕ* ∪ {0} are Adomian's polynomials which can be obtained by using the formula given as in [13]:

(6) $$\begin{array}{c} A_{n}=\frac{1}{n!}\frac{d^{n}}{d\gamma^{n}}{\left[N\left(\overset{\infty }{\underset{k=0}{\sum }}y_{k}\gamma^{k}\right)\right]}_{\gamma =0},\;n=0,1,2,\ldots . \end{array}$$

Recently, El-Kalla [16] proposed another programmable formula for Adomian's polynomials:

(7) $$\begin{array}{c} A_{n}=N\left(\phi_{n}\right)-\overset{n-1}{\underset{j=0}{\sum }}A_{j}\\ \;\text{or}\;N\left(\phi_{n}\right)=\overset{n}{\underset{j=0}{\sum }}A_{j}, \end{array}$$

where *ϕ*_*n*_ = ∑_*j*=0_^*n*^ *y*_*j*_ is the partial sum of the series solution *y* = ∑_*j*=0_^*∞*^ *y*_*j*_.

Substituting the series (4) and (5) into (3), we obtain

(8) $$\begin{array}{c} \overset{\infty }{\underset{j=0}{\sum }}y_{j}\left(x\right)=f\left(x\right)+\overset{b}{\underset{a}{\int }}\left[\overset{\infty }{\underset{j=0}{\sum }}A_{j}\right]dt. \end{array}$$

Comparing both sides of (8), we obtain the ADM scheme:

(9) $$\begin{array}{c} y_{0}\left(x\right)=f\left(x\right),\;\;y_{j}\left(x\right)=\overset{b}{\underset{a}{\int }}A_{j-1}dt,\;j\geq 1. \end{array}$$

Using the recursive scheme (9), the *n*-term approximate series solution can be obtained as follows:

(10) $$\begin{array}{c} \phi_{n}\left(x\right)=\overset{n}{\underset{j=0}{\sum }}y_{j}\left(x\right). \end{array}$$

Now we discuss the convergence analysis and error bounds for the recursive scheme (9). Let *𝕏* = *C* [0,1] be the Banach space with the norm ||*y*|| = max_*x*∈*Ω*_ | *y*(*x*)|. Note that (3) can be written in the operator as

(11) $$\begin{array}{c} y=𝒩\left(y\right), \end{array}$$

where *𝒩*(*y*) is given by

(12) $$\begin{array}{c} 𝒩\left(y\right)=f\left(x\right)+\overset{b}{\underset{a}{\int }}K\left(x,t,y\left(t\right)\right)dt. \end{array}$$

We next discuss the existence of the unique solution of (11).

**Theorem 1 (contraction principle)**

Let *𝕏* be the Banach space with the norm ||*y*|| = max_*x*∈*Ω*_ | *y*(*x*)|. Assume that the nonlinear kernel *K* (*x*, *t*, *y*) satisfies the Lipschitz condition such that

(13) $$\begin{array}{c} \left|K\left(x,t,y\right)-K\left(x,t,y^{*}\right)\right|\leq L\left|y-y^{*}\right|. \end{array}$$

Further, one defines *δ* : = *L* (*b* − *a*), where *L* is Lipschitz constant. If *δ* < 1, then the solution of (11) exists and unique.

**Proof**

For any *y*, y* ∈ *𝕏* and using Lipschitz continuity of *K* (*x*, *t*, *y*) as defined in (2), we have

(14) ||𝒩(y)−𝒩(y∗)||  =maxx∈Ω|∫ab[K(x,t,y(t))−K(x,t,y∗(t))]dt|,  ≤maxx∈Ω L(b−a)|y(t)−y∗(t)|.

Thus we have

(15) $$\begin{array}{c} ||𝒩\left(y\right)-𝒩\left(y^{*}\right)||\leq \delta ||y-y^{*}||, \end{array}$$

where *δ* = *L* (*b* − *a*). If *δ* < 1, then *𝒩* : *𝕏* → *𝕏* is contraction and hence, by the Banach contraction mapping theorem, (11) has a unique solution in *𝕏*.

Now we write scheme (9) in operator form as follows. Let {*ϕ*_*n*_} be the sequence of partial sums of the series solution ∑_*j*=0_^*∞*^ *y*_*j*_.

By using the recursive scheme (9) and (10), we have

(16) ϕn=y0+∑j=1nyj=f(x)+∑j=1n[∫abAj−1dt]=f(x)+∫ab[∑j=0n−1Aj]dt.

Using (7) into (16), it follows that

(17) $$\begin{array}{c} \phi_{n}=f\left(x\right)+\overset{b}{\underset{a}{\int }}K\left(x,t,\phi_{n-1}\right)dt, \end{array}$$

which is equivalent to the following operator equation:

(18) $$\begin{array}{c} \phi_{n}=𝒩\left(\phi_{n-1}\right),\;n=1,2,\ldots . \end{array}$$

In the next theorem, we discuss the convergence of the approximate series solution *ϕ*_*n*_ to the exact solution *y*.

**Theorem 2**

Let *𝒩* be the nonlinear operator defined by (12) contractive; that is,

(19) ||𝒩(y)−𝒩(y∗)||≤δ||y−y∗||,∀y,y∗∈𝕏  with  δ<1. 

If ||*y*_1_|| < *∞*, then the sequence {*ϕ*_*n*_} defined by (18) converges to the exact solution *y*.

**Proof**

Using relation (18) and the fact that *𝒩* is contractive, we have

(20) ||ϕm+1−ϕm||=||𝒩(ϕm)−𝒩(ϕm−1)||≤δ||ϕm−ϕm−1||.

Thus we have

(21) ||ϕm+1−ϕm||≤δ||ϕm−ϕm−1||≤δ2||ϕm−1−ϕm−2||≤⋯≤δm||ϕ1−ϕ0||.

Now for all *n*, *m* ∈ *ℕ*, with *n* ≥ *m*, consider

(22) ||ϕn−ϕm||  =||(ϕn−ϕn−1)+(ϕn−1−ϕn−2)+⋯+(ϕm+1−ϕm)||  ≤||ϕn−ϕn−1||+||ϕn−1−ϕn−2||+⋯+||ϕm+1−ϕm||  ≤[δn−1+δn−2+⋯+δm]||ϕ1−ϕ0||  =δm[1+δ+δ2+⋯+δn−m−1]||ϕ1−ϕ0||  =δm(1−δn−m1−δ)||y1||.

Since 0 < *δ* < 1, so, (1 − *δ* ^*n*−*m*^) < 1, and ||*y*_1_|| < *∞*, it follows that

(23) $$\begin{array}{c} ||\phi_{n}-\phi_{m}||\leq \frac{\delta^{m}}{1-\delta }||y_{1}|| \end{array}$$

which converges to zero; that is, ||*ϕ*_*n*_ − *ϕ*_*m*_|| → 0, as *m* → *∞*. This implies that there exists *ϕ* such that lim_*n*→*∞*_ *ϕ*_*n*_ = *ϕ*. Since, we have *y* = ∑_*j*=0_^*∞*^ *y*_*j*_ = lim_*n*→*∞*_ *ϕ*_*n*_, that is, *y* = *ϕ* which is exact solution of (11).

In the following theorem we obtain the error bounds for the approximate series solution *ϕ*_*n*_.

**Theorem 3**

Let *y* be the exact solution of (11). Let *ϕ*_*m*_ be the sequence of approximate series solution given by (18). Then there holds

(24) $$\begin{array}{c} ||y-\phi_{m}||\leq \frac{\left(b-a\right)\delta^{m}}{\left(1-\delta \right)}\underset{\left(x,t\right)\in \Omega \times \Omega }{\max}\left|K\left(x,t,y_{0}\right)\right|. \end{array}$$

**Proof**

From estimate (23), for *n* ≥ *m*, *n*, *m* ∈ *ℕ*, we have

(25) $$\begin{array}{c} ||\phi_{n}-\phi_{m}||\leq \frac{\delta^{m}}{1-\delta }||y_{1}||. \end{array}$$

Since lim_*n*→*∞*_ *ϕ*_*n*_ = *y*, fixing *m* and letting *n* → *∞*, we obtain

(26) $$\begin{array}{c} ||y-\phi_{m}||\leq \frac{\delta^{m}}{1-\delta }\underset{x\in \Omega }{\max}\left|y_{1}\left(x\right)\right|. \end{array}$$

Since *y*_1_(*x*) = ∫_*a*_^*b*^ *A*_0_ *dξ* and *A*_0_ = *K* (*x*, *t*, *y*_0_), we have

(27) maxx∈Ω|y1(x)|=maxx∈Ω|∫abK(x,t,y0)dt|≤(b−a)max(x,t)∈Ω×Ω|K(x,t,y0)|.

Combining estimates (26) and (27), we obtain

(28) $$\begin{array}{c} ||y-\phi_{m}||\leq \frac{\left(b-a\right)\delta^{m}}{\left(1-\delta \right)}\underset{(x,t)\in \Omega \times \Omega }{\max}\left|K\left(x,t,y_{0}\right)\right|. \end{array}$$

This completes the proof.

## 3. Numerical Results

In this section, we apply scheme (9) to solve three examples. All numerical results obtained by ADM are compared with known results and with those in [5].

**Example 4**

Consider the following nonlinear Fredholm integral equation Urysohn form [5]:

(29) $$\begin{array}{c} y\left(x\right)=f\left(x\right)+\overset{1}{\underset{0}{\int }}K\left(x,t,y\left(t\right)\right)dt,\;0\leq x\leq 1, \end{array}$$

with *f*(*x*) = sin*πx* and *K* (*x*, *t*, *y*(*t*)) = (1/5)cos*πx*sin*πty* ^3^(*t*). Its analytical solution is given by $y(x)=\sin\pi x+(1/3)(20-\sqrt{391})\cos\pi x$.

According to the ADM (9), the problem (29) is transformed into the following recursive scheme:

(30) $$\begin{array}{c} y_{0}\left(x\right)=\sin\pi x,\\ y_{j}\left(x\right)=\frac{1}{5}\overset{1}{\underset{0}{\int }}\cos\pi x\sin\pi tA_{j-1}dt,\;j\geq 1. \end{array}$$

The Adomian's polynomials *A*_*j*_ are computed for *y* ^3^(*x*) by using formula (6):

(31) $$\begin{array}{c} A_{0}=y_{0}^{3}\left(x\right),\;\;A_{1}=3y_{0}^{2}\left(x\right)y_{1}\left(x\right),\\ A_{2}=3y_{0}\left(x\right)y_{1}^{2}\left(x\right)+3y_{0}^{2}\left(x\right)y_{1}\left(x\right),\ldots \end{array}$$

For quantitative comparison, we define the absolute error functions as

(32) $$\begin{array}{c} e_{n}\left(x\right)=\left|\phi_{n}\left(x\right)-y\left(x\right)\right|, \end{array}$$

where *y*(*x*) is analytical solution and *ϕ*_*n*_(*x*) is *n*-term truncated series solutions obtained by ADM (9).  Table 1 shows the comparison of the numerical results obtained by the present recursive (9) and Newton-Kantorovich-quadrature used in [5]. One can note that the present method gives much better numerical results compared to Newton-Kantorovich-quadrature method.

**Example 5**

We now consider the following nonlinear Urysohn Volterra-integral equation [5]:

(33) $$\begin{array}{c} y\left(x\right)=f\left(x\right)+\overset{x}{\underset{0}{\int }}K\left(x,t,y\left(t\right)\right)dt,\;0\leq x\leq 1, \end{array}$$

with *f* (*x*) = sin*x* − (*x*/2)+(1/4)sin2*x* and *K* (*x*, *t*, *y*(*t*)) = *y* ^2^(*t*). The exact solution to this integral equation is *y* (*x*) = sin*x*.

According to the ADM (9), the problem (33) is transformed into the following recursive scheme:

(34) $$\begin{array}{c} y_{0}\left(x\right)=\sin x-\frac{x}{2}+\frac{1}{4}\sin2x,\\ y_{j}\left(x\right)=\overset{x}{\underset{0}{\int }}A_{j-1}dt,\;j\geq 1. \end{array}$$

Using formula (6), we obtain the Adomian's polynomials for *y* ^2^(*x*) as follows:

(35) $$\begin{array}{c} A_{0}=y_{0}^{2}\left(x\right),\;\;A_{1}=2y_{0}\left(x\right)y_{1}\left(x\right),\\ A_{2}=2y_{0}\left(x\right)y_{2}\left(x\right)+y_{1}^{2}\left(x\right),\ldots \end{array}$$

**Figure 1:**
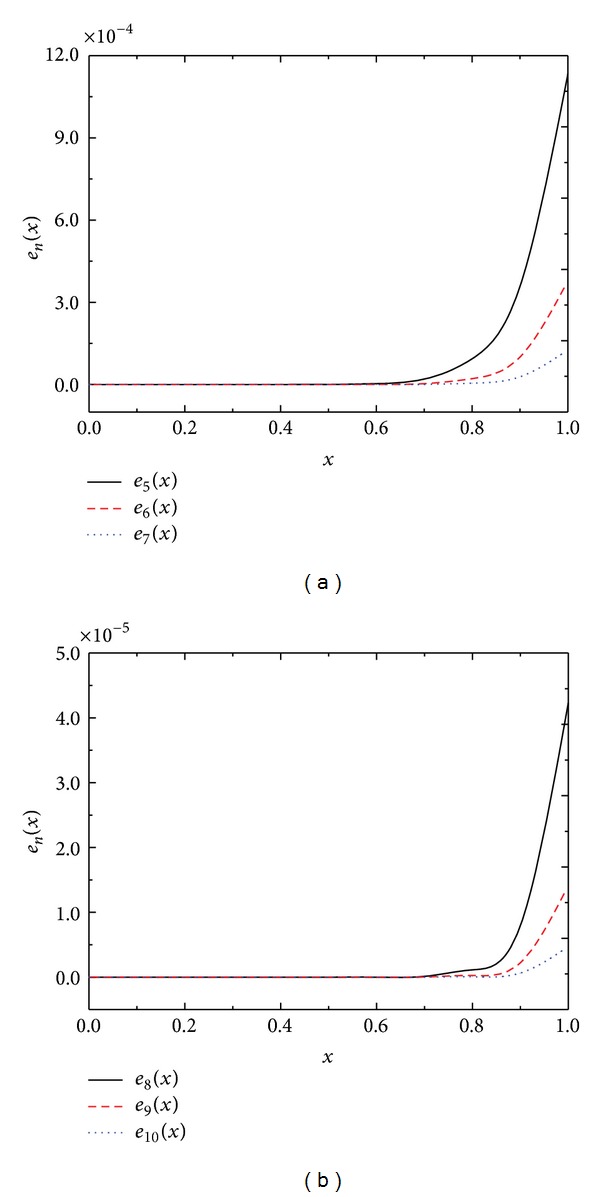
Absolute error functions *e*_*n*_(*x*) of Example 5.

Once again, we compare the numerical results obtained by the present recursive (9) and Newton-Kantorovich-quadrature used in [5] in Table 2. We again conclude that the present method gives much better numerical results compared to Newton-Kantorovich-quadrature method [5]. Furthermore, we also plot absolute error function *e*_*n*_, *n* = 5,6,…, 10 in Figures 1(a) and 1(b). From these figures we can clearly see that as the iterations increase the error decreases.

**Example 6**

Finally, we consider the following nonlinear Urysohn integral equation [8]:

(36) y(x)=f(x)+∫01K(x,t,y(y))dt,where  K1(x,t)={x(t−1),x≤t,t(x−1),t≤x,

with *f*(*x*) = 0 and *K* (*x*, *t*, *y*(*t*)) = *K*_1_ (*x*, *t*)*e* ^*y*(*t*)^. The exact solution is given by *y* (*x*) = −ln2 + 2ln(*c*/cos[*c*(*x* − 1/2)/2]), where *c* is the solution of $c-\sqrt{2}\cos(c/4)=0$.

According to the ADM (9), the problem (31) is transformed into the following recursive scheme:

(37) $$\begin{array}{c} y_{0}\left(x\right)=0,\;\;y_{j}\left(x\right)=\overset{1}{\underset{0}{\int }}K\left(x,t\right)A_{j-1}dt,\;j\geq 1. \end{array}$$

Using formula (6), we obtain the Adomian's polynomials for *e* ^*y*(*x*)^ as follows

(38) $$\begin{array}{c} A_{0}=e^{y_{0}(x)},\;\;A_{1}=e^{y_{0}(x)}y_{1}x,\\ A_{2}=e^{y_{0}(x)}\left(\frac{1}{2}y_{1}^{2}\left(x\right)+y_{2}\left(x\right)\right),\ldots \end{array}$$

**Figure 2:**
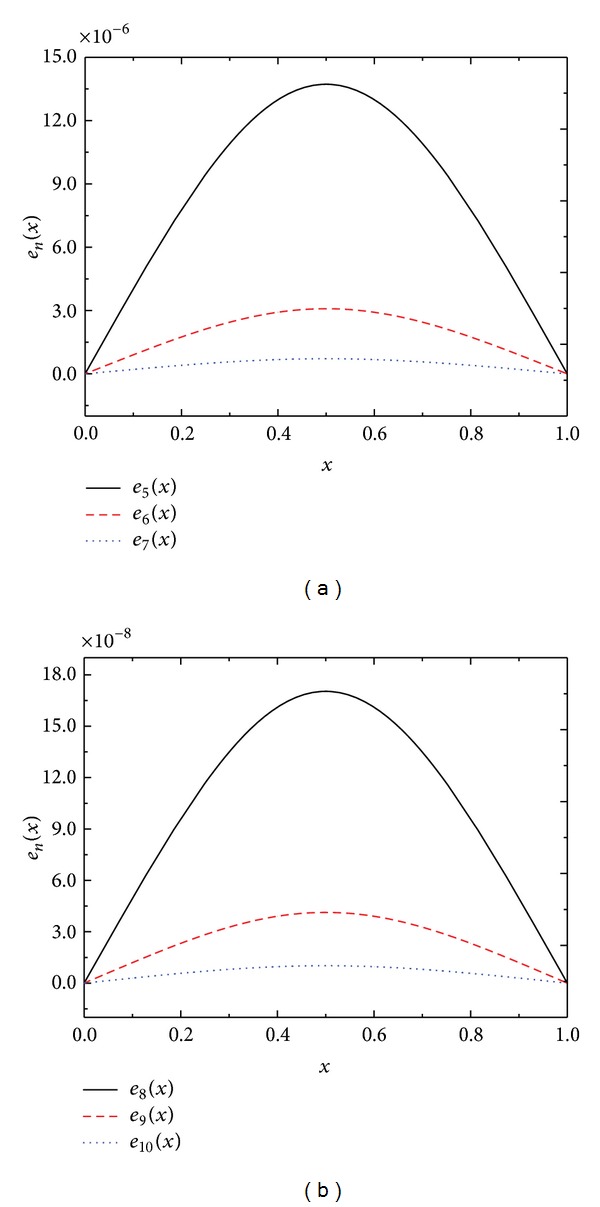
Absolute error functions *e*_*n*_(*x*) of Example 6.

Like previous examples, Table 3 shows the numerical results obtained by present method (9). In addition, we also plot absolute error functions *e*_*n*_ for *n* = 5,6,…, 10 in Figures 2(a) and 2(b), and it is shown that only a few terms are required to obtain acceptable approximation for the solution.

## 4. Conclusion

In this paper, we have applied the ADM to solve nonlinear Urysohn type integral equation. The accuracy of the numerical results indicates that the proposed method is well suited for the solution of such type of problems. The advantage of current approach is that it provides a direct scheme for obtaining approximations of the solutions. Moreover, the proposed method provides a reliable technique which requires less work compared to the traditional techniques such as finite difference method (FDM), cubic spline method (CSM), B-spline method (BSM), and Newton-Kantorovich-quadrature method. Unlike FDM, CSM, and any other discrete methods, the ADM does not require any linearization or discretization of variables. The numerical results show that only a few terms are required to obtain accurate solutions. By comparing the numerical results with other existing method used in [5], it has been shown that proposed method is a powerful method for solving nonlinear Urysohn integral equations. Finally, we have also discussed the convergence and error analysis of the ADM.

## Figures and Tables

**Table 1:** Numerical solution and absolute error of Example 4.

*x*	Exact	*ϕ*_10_	|*ϕ*_1_ − *y*|	|*ϕ*_3_ − *y*|	Error [5]
0.0	0.075426689	0.075426689	4.2668*E* − 04	4.8139*E* − 06	4.9831*E* − 02
0.1	0.380752038	0.380752038	4.0580*E* − 04	4.5783*E* − 06	4.7392*E* − 02
0.2	0.648806725	0.648806725	3.4519*E* − 04	3.8945*E* − 06	4.0314*E* − 02
0.3	0.853351690	0.853351690	2.5080*E* − 04	2.8295*E* − 06	2.9290*E* − 02
0.4	0.974364645	0.974364645	1.3185*E* − 04	1.4875*E* − 06	1.5398*E* − 02
0.5	1.000000000	1.000000000	0.0000*E* − 00	0.0000*E* − 00	0.0000*E* − 00
0.6	0.927748388	0.927748388	1.3185*E* − 04	1.4875*E* − 06	1.5398*E* − 02
0.7	0.764682299	0.764682299	2.5080*E* − 04	2.8295*E* − 06	2.9290*E* − 02
0.8	0.526763779	0.526763779	3.4519*E* − 04	3.8945*E* − 06	4.0314*E* − 02
0.9	0.237281950	0.237281950	4.0580*E* − 04	4.5783*E* − 06	4.7392*E* − 02
1.0	−0.075426689	−0.075426689	4.2668*E* − 04	4.8139*E* − 06	4.9831*E* − 02

**Table 2:** Numerical solution and absolute error of Example 5.

*x*	Exact	*ϕ*_10_	|*ϕ*_2_ − *y*|	|*ϕ*_4_ − *y*|	Error [5]
0.0	0.000000000	0.000000000	0.0000*E* − 00	0.0000*E* − 00	0.0000*E* − 00
0.1	0.099833417	0.099833417	5.3658*E* − 09	8.7652*E* − 14	3.3266*E* − 04
0.2	0.198669331	0.198669331	6.7508*E* − 07	1.7463*E* − 10	1.5351*E* − 03
0.3	0.295520207	0.295520207	1.1207*E* − 05	1.4393*E* − 08	6.6505*E* − 03
0.4	0.389418342	0.389418342	8.0648*E* − 05	3.1851*E* − 07	1.3901*E* − 02
0.5	0.479425539	0.479425539	3.6516*E* − 04	3.3996*E* − 06	2.4222*E* − 02
0.6	0.564642473	0.564642473	1.2282*E* − 03	2.2720*E* − 05	4.6982*E* − 02
0.7	0.644217687	0.644217683	3.3532*E* − 03	1.0927*E* − 04	6.8954*E* − 02
0.8	0.717356091	0.717356031	7.8338*E* − 03	4.1109*E* − 04	9.5915*E* − 02
0.9	0.783326910	0.783326286	1.6204*E* − 02	1.2762*E* − 03	1.4380*E* − 01
1.0	0.841470985	0.841466258	3.0377*E* − 02	3.3927*E* − 03	1.8538*E* − 01

**Table 3:** Numerical solution and absolute error of Example 6.

*x*	Exact	*ϕ*_10_	|*ϕ*_2_ − *y*|	|*ϕ*_4_ − *y*|	|*ϕ*_6_ − *y*|
0.0	1.11022*E* − 16	0.00000*E* − 00	1.1102*E* − 16	1.1102*E* − 16	1.1102*E* − 16
0.1	−0.041435623	−0.041435620	5.2312*E* − 04	1.8827*E* − 05	9.0212*E* − 07
0.2	−0.073268382	−0.073268376	1.0017*E* − 03	3.6337*E* − 05	1.7458*E* − 06
0.3	−0.095799848	−0.095799840	1.3873*E* − 03	5.0792*E* − 05	2.4489*E* − 06
0.4	−0.109237721	−0.109237712	1.6377*E* − 03	6.0385*E* − 05	2.9199*E* − 06
0.5	−0.113703656	−0.113703646	1.7244*E* − 03	6.3752*E* − 05	3.0862*E* − 06
0.6	−0.109237721	−0.109237712	1.6377*E* − 03	6.0385*E* − 05	2.9199*E* − 06
0.7	−0.095799848	−0.095799840	1.3873*E* − 03	5.0792*E* − 05	2.4489*E* − 06
0.8	−0.073268382	−0.073268376	1.0017*E* − 03	3.6337*E* − 05	1.7458*E* − 06
0.9	−0.041435623	−0.041435620	5.2312*E* − 03	1.8827*E* − 05	9.0212*E* − 07
1.0	1.11022*E* − 16	2.88127*E* − 17	9.0205*E* − 17	1.3183*E* − 16	1.2533*E* − 16
